# Genotype-specific effects of elamipretide in patients with primary mitochondrial myopathy: a post hoc analysis of the MMPOWER-3 trial

**DOI:** 10.1186/s13023-024-03421-5

**Published:** 2024-11-21

**Authors:** Amel Karaa, Enrico Bertini, Valerio Carelli, Bruce Cohen, Gregory M. Ennes, Marni J. Falk, Amy Goldstein, Gráinne Gorman, Richard Haas, Michio Hirano, Thomas Klopstock, Mary Kay Koenig, Cornelia Kornblum, Costanza Lamperti, Anna Lehman, Nicola Longo, Maria Judit Molnar, Sumit Parikh, Han Phan, Robert D. S. Pitceathly, Russekk Saneto, Fernando Scaglia, Serenella Servidei, Mark Tarnopolsky, Antonio Toscano, Johan L. K. Van Hove, John Vissing, Jerry Vockley, Jeffrey S. Finman, Anthony Abbruscato, David A. Brown, Alana Sullivan, James A. Shiffer, Michelango Mancuso, on behalf of the MMPOWER-3 Trial Investigators

**Affiliations:** 1 Massachusetts General Hospital, Genetics Division Harvard Medical School Boston, Boston, MA USA; 2 Neuromuscular Unit, Bambino Gesù Ospedale Pediatrico, IRCCS, Rome, Italy; 3 IRCCS Istituto Delle Scienze Neurologiche Di Bologna, Programma Di Neurogenetica, Bologna, Italy; 4 Department of Biomedical and Neuromotor Sciences, University of Bologna, Bologna, Italy; 5 Akron Children’s Hospital, Rebecca D. Considine Research Institute, Akron, OH USA; 6 Stanford University School of Medicine, Stanford, CA USA; 7 Division of Human Genetics, Department of Pediatrics, Children’s Hospital of Philadelphia and University of Pennsylvania Perelman School of Medicine, Mitochondrial Medicine Frontier Program, Philadelphia, PA USA; 8 Royal Victoria Infirmary, Newcastle Upon Tyne, England; 9 University of California, San Diego, La Jolla, CA USA; 10 Columbia University Irving Medical Center, New York, NY USA; 11 Department of Neurology, LMU Hospital, Friedrich-Baur-Institute, Ludwig-Maximilians-Universität Munich, Munich, Germany; 12 German Center for Neurodegenerative Diseases (DZNE), Munich, Germany; 13 Munich Cluster for Systems Neurology (SyNergy), Munich, Germany; 14 Department of Pediatrics, Division of Child and Adolescent Neurology, Center for the Treatment of Pediatric Neurodegenerative Disease, University of Texas McGovern Medical School, Houston, TX USA; 15 Department of Neurology, University Hospital of Bonn, Neuromuscular Diseases Section, Bonn, Germany; 16 Fondazione IRCCS Istituto Neurologico Carlo Besta, Milan, Italy; 17 Vancouver General Hospital, Vancouver, BC Canada; 18 University of Utah, Salt Lake City, UT USA; 19 Institute of Genomic Medicine and Rare Disorders, Semmelweis University, Budapest, Hungary; 20 Cleveland Clinic Neurological Institute, Cleveland, OH USA; 21 Rare Disease Research, Atlanta, GA USA; 22 Department of Neuromuscular Diseases, UCL Queen Square Institute of Neurology, London, UK; 23 NHS Highly Specialised Service for Rare Mitochondrial Disorders, Queen Square Centre for Neuromuscular Diseases, The National Hospital for Neurology and Neurosurgery, London, UK; 24 Seattle Children’s Hospital, Seattle, WA USA; 25 Department of Molecular and Human Genetics, Baylor College of Medicine, Houston, TX USA; 26 Texas Children’s Hospital, Houston, TX USA; 27 Joint BCM-CUHK Center of Medical Genetics, Prince of Wales Hospital, Sha Tin, Hong Kong SAR, China; 28 Fondazione Policlinico Universitario A. Gemelli and Istituto Di Neurologia, Università Cattolica del Sacro Cuore, Rome, Italy; 29 Division of Neuromuscular and Neurometabolic Disorders, McMaster University Children’s Hospital, Hamilton, ON Canada; 30 Department of Clinical and Experimental Medicine, ERN-NMD Center for Neuromuscular Disorders of Messina, University of Messina, Messina, Italy; 31 University of Colorado and Children’s Hospital Colorado, Aurora, CO USA; 32 Copenhagen Neuromuscular Center, Rigshospitalet, University of Copenhagen, Copenhagen, Denmark; 33 Children’s Hospital of Pittsburgh, University of Pittsburgh School of Medicine, Pittsburgh, PA USA; 34 Jupiter Point Pharma Consulting, LLC, Jupiter, CT USA; 35 Stealth BioTherapeutics, Needham, MA USA; 36 Write On Time Medical Communications, LLC, Medford, NJ USA; 37 Department of Clinical and Experimental Medicine, Neurological Institute, University of Pisa, Pisa, Italy

**Keywords:** Elamipretide, PMM, Replisome, Mitochondria, MtDNA maintenance, MtDNA multiple deletions

## Abstract

**Background:**

As previously published, the MMPOWER-3 clinical trial did not demonstrate a significant benefit of elamipretide treatment in a genotypically diverse population of adults with primary mitochondrial myopathy (PMM). However, the prespecified subgroup of subjects with disease-causing nuclear DNA (nDNA) pathogenic variants receiving elamipretide experienced an improvement in the six-minute walk test (6MWT), while the cohort of subjects with mitochondrial DNA (mtDNA) pathogenic variants showed no difference versus placebo. These published findings prompted additional genotype-specific post hoc analyses of the MMPOWER-3 trial. Here, we present these analyses to further investigate the findings and to seek trends and commonalities among those subjects who responded to treatment, to build a more precise Phase 3 trial design for further investigation in likely responders.

**Results:**

Subjects with mtDNA pathogenic variants or single large-scale mtDNA deletions represented 74% of the MMPOWER-3 population, with 70% in the mtDNA cohort having either single large-scale mtDNA deletions or *MT-TL1* pathogenic variants. Most subjects in the nDNA cohort had pathogenic variants in genes required for mtDNA maintenance (mtDNA replisome), the majority of which were in *POLG* and *TWNK*. The mtDNA replisome *post-hoc* cohort displayed an improvement on the 6MWT, trending towards significant, in the elamipretide group when compared with placebo (25.2 ± 8.7 m versus 2.0 ± 8.6 m for placebo group; *p* = 0.06). The 6MWT results at week 24 in subjects with replisome variants showed a significant change in the elamipretide group subjects who had chronic progressive external ophthalmoplegia (CPEO) (37.3 ± 9.5 m versus − 8.0 ± 10.7 m for the placebo group; *p* = 0.0024). Pharmacokinetic (exposure–response) analyses in the nDNA cohort showed a weak positive correlation between plasma elamipretide concentration and 6MWT improvement.

**Conclusions:**

Post hoc analyses indicated that elamipretide had a beneficial effect in PMM patients with mtDNA replisome disorders, underscoring the importance of considering specific genetic subtypes in PMM clinical trials. These data serve as the foundation for a follow-up Phase 3 clinical trial (NuPOWER) which has been designed as described in this paper to determine the efficacy of elamipretide in patients with mtDNA maintenance-related disorders.

**Classification of evidence:**

Class I

**ClinicalTrials.gov identifier:**

NCT03323749

## Background

As a diverse group of genetically confirmed disorders, primary mitochondrial myopathies (PMMs) predominantly, but not exclusively, affect skeletal muscle, adversely impacting physical function and quality of life [1]. Although individual mitochondrial diseases are rare, PMMs are a common manifestation of primary mitochondrial diseases, with an estimated prevalence of 1–2 in 10,000 [2, 3]. PMM patients often display muscular weakness, muscle atrophy, limited exercise capacity, and fatigue [1, 4, 5], with no currently approved therapies.

The largest Phase 3 clinical trial to date in patients with PMM, the MMPOWER3 trial, was recently completed [6]. This trial evaluated the efficacy and safety of daily elamipretide, a mitochondria-targeting peptide, as a treatment for patients with genetically confirmed PMM [6]. The trial enrolled a highly heterogeneous population of myopathic patients with a variety of pathogenic variants in either nuclear (nDNA) or mitochondrial (mtDNA) genes [6]. Mitochondria require the coordinated translation of genes encoded by both nDNA and mtDNA, and PMMs can be caused by alterations in either genome. mtDNA encodes a handful of lipophilic electron transport chain subunits, and ribosomal/transfer RNAs used in mtDNA translation. Almost all (~ 99%) of the mitochondrial proteome is encoded by nDNA, including all proteins responsible for replicating mtDNA (the mtDNA replisome). Alterations in these proteins, caused by nuclear gene defects, are collectively referred to as mtDNA maintenance disorders, or mtDNA depletion and deletions syndrome (MDDS), with myopathy being a common clinical occurrence [7].

Although MMPOWER-3 did not meet its primary endpoints assessing changes in the Six-Minute Walk Test (6MWT) and fatigue in the total population, a post hoc subgroup analysis revealed that subjects with nDNA pathogenic variants experienced an improvement in 6MWT compared with placebo [6]. Based on these findings, further in-depth analysis was warranted to better understand the genotype-specific responses in the trial, and to enhance the likelihood of success for future clinical trials in individuals with nuclear primary mitochondrial disease (nPMD).

## Methods

### Trial design

Full details of MMPOWER-3 have been previously described [6]. In brief, MMPOWER-3 was a 24-week, randomized (1:1), double-blind, parallel-group, placebo-controlled clinical trial for adult patients with PMM, in which subjects received elamipretide 40 mg subcutaneously once daily or placebo [6]. In the original analysis of MMPOWER-3, subjects were stratified by the type of pathogenic DNA variant (nDNA vs mtDNA) determined to be the primary cause of PMM as approved by the adjudication committee [6]. Pathogenic DNA variants causing PMM were subclassified as causing mtDNA or nDNA disorders [6]. The prespecified exploratory analysis was conducted to further examine the effects of elamipretide on the change from baseline to week 24 in the 6MWT by genetic subgroups. Subject demographics at baseline have been previously published in detail [6].

### Standard protocol approvals, registrations, and patient consents

MMPOWER-3 was conducted in accordance with international ethics guidelines, including the Declaration of Helsinki, Council for International Organizations of Medical Sciences International Ethical Guidelines, ICH GCP guidelines, and all applicable laws and regulations [6]. The trial was approved by institutional review boards, and all subjects provided written informed consent [6].

### Statistical analysis

In the original analysis of MMPOWER-3, the efficacy of elamipretide was analyzed by genetic pathogenic variant subclass (mtDNA vs. nDNA) utilizing a mixed model repeated measures (MMRM) [6]. In the new exploratory analysis, the effect of elamipretide on the least squares (LS) mean change from baseline in distance walked on the 6MWT at 4 weeks, 12 weeks, and end of treatment (week 24) was examined as a function of gene variants using subjects from the MMPOWER-3 per-protocol population who successfully completed the trial. The analysis evaluated 6MWT results by specific mtDNA and nDNA genotypes. Efficacy in the mtDNA replisome subgroup was further assessed by the presence of the chronic progressive external ophthalmoplegia (CPEO) as a phenotype.

A pharmacokinetic/pharmacodynamic analysis was also performed in the nDNA population to assess the absolute change in the 6MWT as a function of steady-state elamipretide area under the plasma concentration–time curve (AUC). Regression analysis, with corresponding r (correlation coefficient) and *p* values, and Loess smoothing were performed [8].

## Results

### Genetic subtype data

**Fig. 1:**
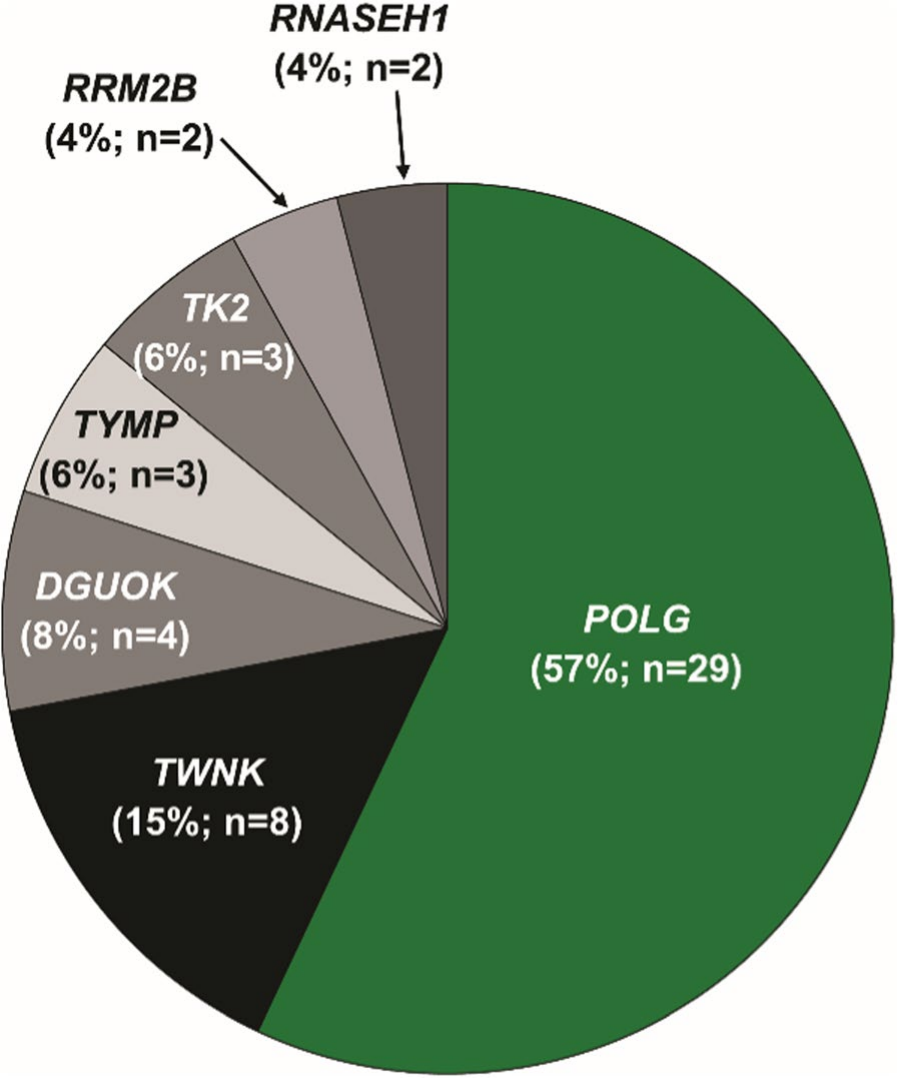
Genotype breakdown of the mtDNA Replisome cohort from MMPOWER-3 (percentage of the cohort [N = 51])

The mtDNA and nDNA variants within the entire trial population, as well as the finding that subjects with nDNA pathogenic variants who received elamipretide performed significantly better on the 6MWT compared with placebo, have previously been published [6]. Among the nDNA cohort, almost all subjects had pathogenic variants associated with mtDNA maintenance, depicted in Fig. 1. Most of these subjects had *POLG* pathogenic variants, followed by pathogenic variants in *TWNK* that encodes the mtDNA helicase Twinkle, and a handful of other genes encoding replisome-related enzymes, including *DGUOK*, *TYMP*, *TK2*, *RRM2B*, *RNASEH1* (see Fig. 1).

**Fig. 2:**
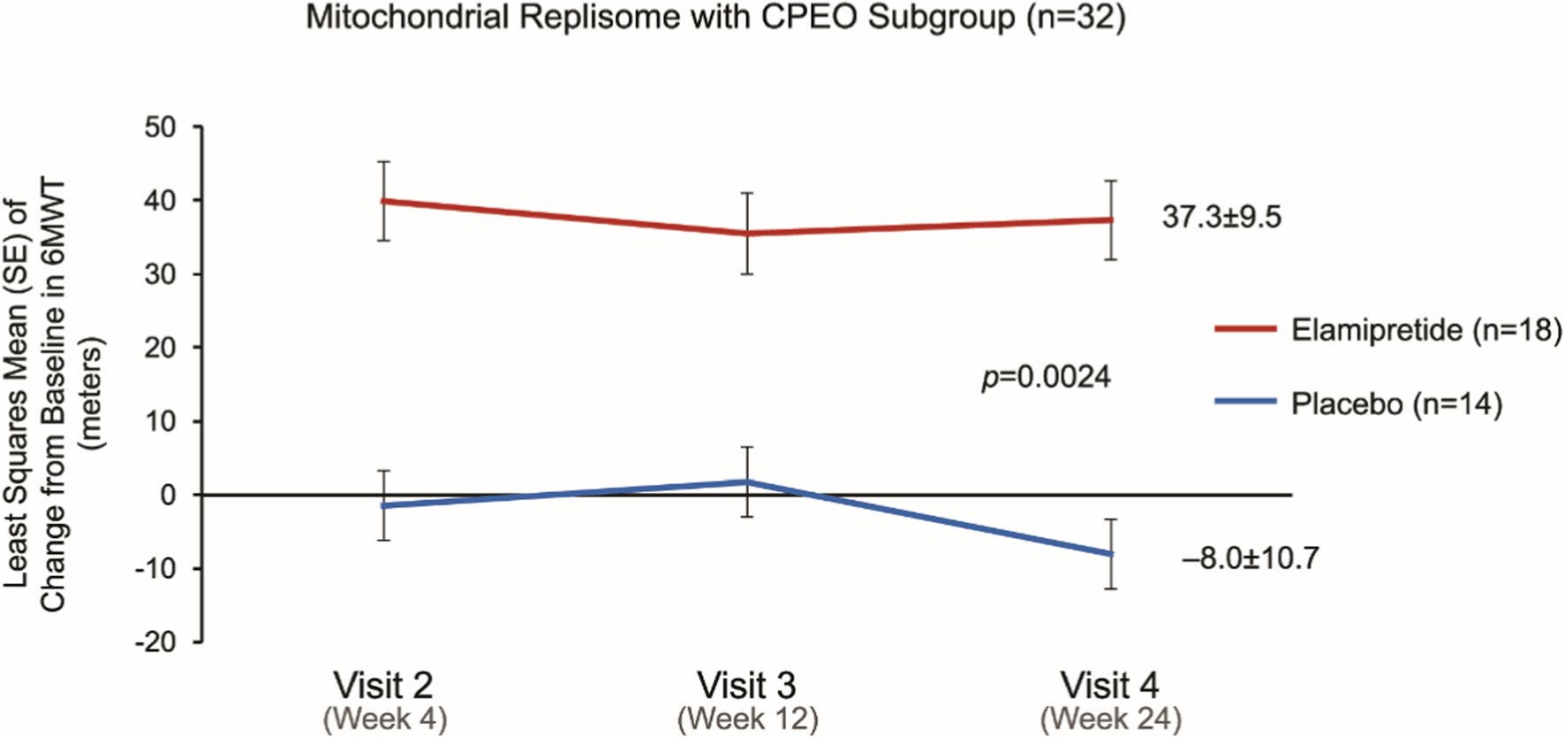
6MWT change from baseline (subgroup replisome pathogenic variants and chronic progressive external ophthalmoplegia [CPEO]) phenotype. 6MWT, 6-min Walk Test; CPEO, chronic progressive external ophthalmoplegia; mtDNA, mitochondrial DNA; nDNA, nuclear DNA

As was previously published [6], in a *post-hoc* analysis, the nDNA cohort (n = 59) displayed a significantly greater improvement in the 6MWT between elamipretide and placebo (25.2 m versus 0.3 m, respectively, *p* = 0.03). The most robust of improvements, however, was observed in the *post-hoc* cohort of subjects who had an mtDNA replisome genotype and a CPEO phenotype (Fig. 2). Subjects with CPEO experienced ptosis, ophthalmoplegia, fatigue and some also exhibited proximal muscle weakness. Baseline functional characteristics of these patients is described elsewhere [6]. At week 24, subjects in the replisome CPEO subgroup who received elamipretide (n = 18) experienced a mean increase from a baseline (mean of 316.5 ± 17.5) of 37.3 ± 9.5 m in the 6MWT, compared with a mean decrease from baseline (324.0 ± 23.4) of − 8.0 ± 10.7 m for the placebo group (n = 14) (*p* = 0.0024).

**Fig. 3:**
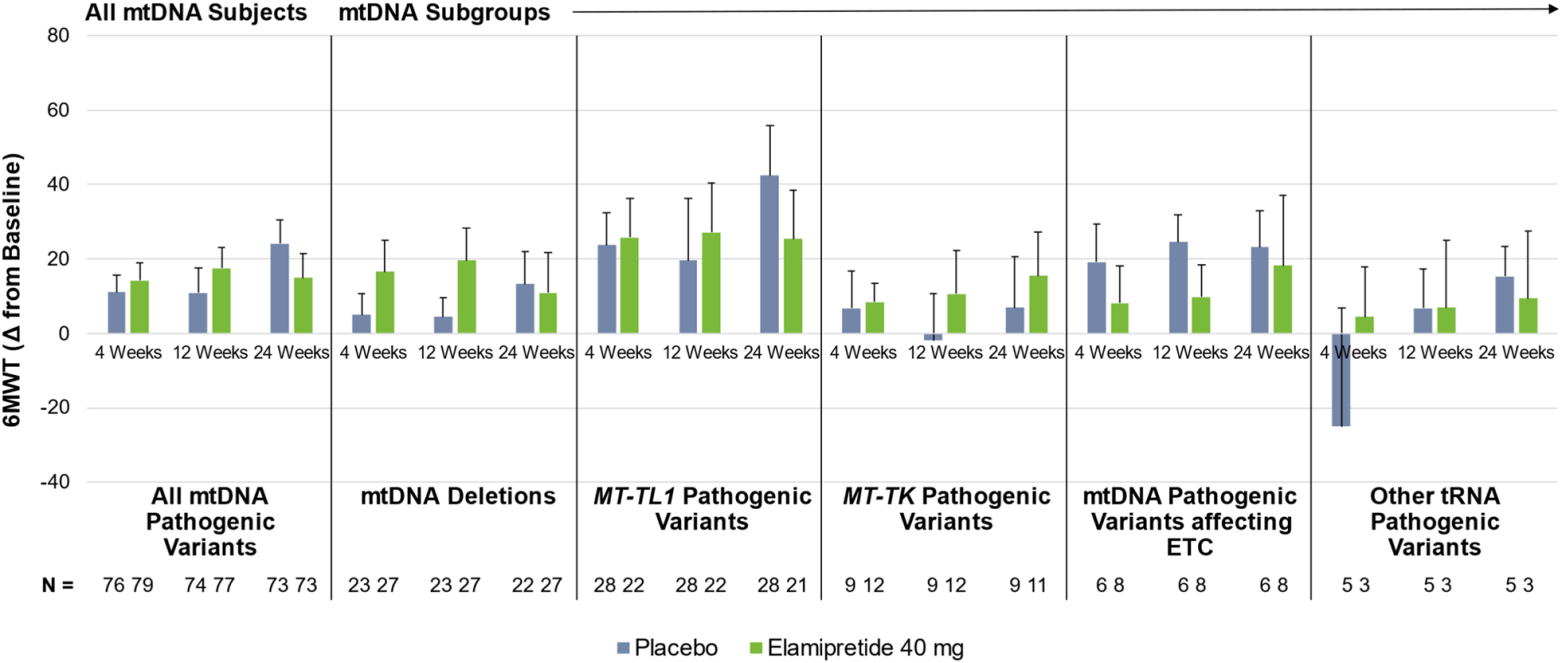
6MWT Change from baseline in the overall mtDNA population and among the mtDNA subgroups. Other tRNA pathogenic variants, as depicted in the graph on the far right, included those found in the transfer tRNAs that encode for the following amino acids: tyrosine (Y), valine (V), glutamic acid (E), isoleucine (I), serine (S), and threonine (T). ETC, electron transport chain; 6MWT, 6-Minute Walk Test; mtDNA, mitochondrial DNA; tRNA, transfer RNA

**Fig. 4:**
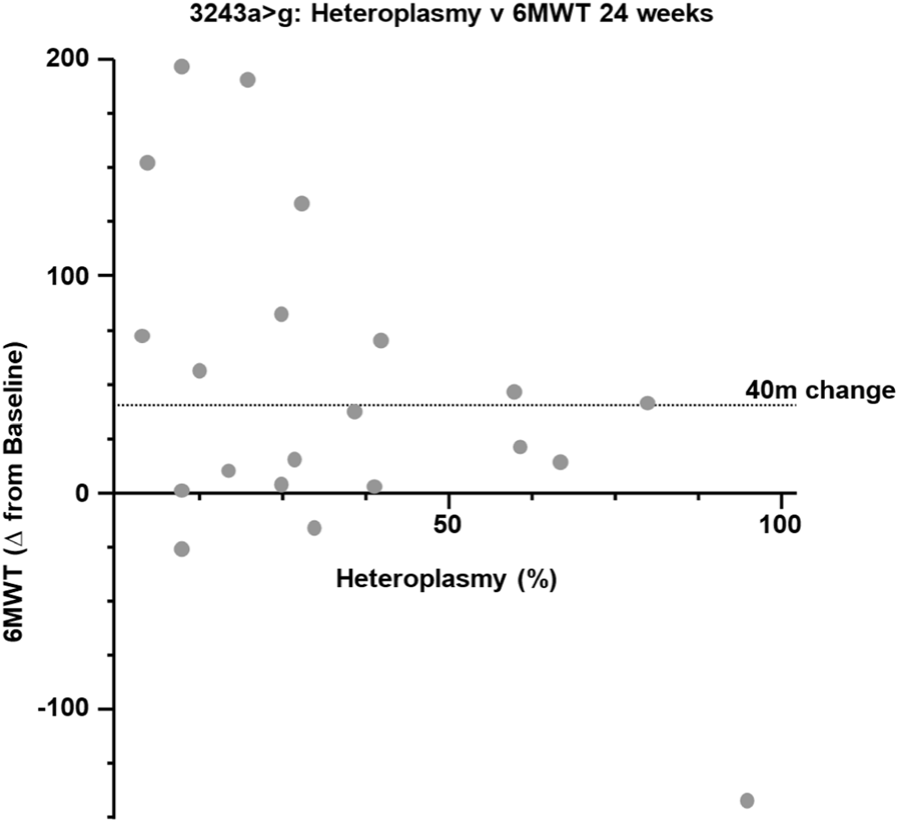
Effect of low heteroplasmy in MT-TL1 placebo subjects on 6MWT

The analysis conducted in this trial also increased understanding of genotype differences relating to elamipretide response within the mtDNA population, as presented in Fig. 3. Here, in this *post-hoc* analysis, the Least Square Means (LS Means) standard error (SE) change from baseline in distance walked on the 6MWT at week 24 was 14.9 ± 6.4 m in subjects with mtDNA pathogenic variants who received elamipretide (n = 73) and 24.1 ± 6.3 m for patients receiving placebo (n = 73), representing a 9.2 m between-group difference in favor of placebo. The difference in favor of placebo was heavily influenced by individuals with *MT-TL1* pathogenic variants (week 24, n = 49). In this cohort, placebo-treated subjects (n = 28) experienced a mean improvement of 42.4 m in the 6MWT compared to baseline (subjects receiving elamipretide [n = 21] walked 25.3 m greater at 24 weeks compared to baseline) (see Fig. 3). Individuals with low heteroplasmy in *MT-TL1* pathogenic variants trended towards having walked significantly farther at week 24 (Fig. 4). Given the high number of individuals in the trial with *MT-TL1* pathogenic variants, this placebo effect heavily influenced the overall results of the MMPOWER-3 Phase 3 trial. Individuals with single mtDNA deletions (week 24, n = 49) also represented a large portion of the mtDNA cohort (week 24, n = 146), with no observable differences at week 24 between elamipretide and placebo-treated subjects.

**Fig. 5:**
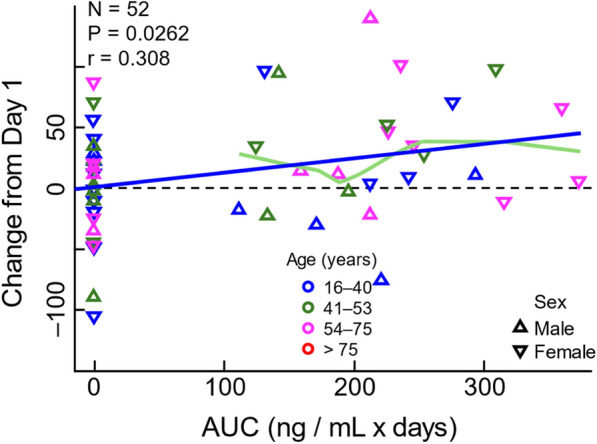
Exposure–response analysis (nDNA cohort at week 24). Change in 6MWT nDNA pathogenic variants as a function of elamipretide steady-state AUC. Placebo subjects are shown with AUC = 0. Symbols indicate sex; colors indicate age bracket. A regression line (and the corresponding P and r values) and a smoother (Loess) are displayed for the elamipretide group. The green smoother excludes values below the limit of quantification

Considering the encouraging signal seen in the nDNA cohort, we conducted exposure–response regression analyses to better understand the pharmacokinetic-pharmacodynamic relationship from the Phase 3 trial. These data are presented in Fig. 5. There was a weak correlation between plasma elamipretide exposure (expressed as AUC) and 6MWT improvement in this cohort when evaluated as the change from baseline to Week 24 (r = 0.308; *p* = 0.0262).

## Discussion

Elamipretide is the first experimental therapeutic compound progressing to a Phase 3 clinical trial in patients with PMM (MMPOWER-3) [6]. This trial followed the Phase 1/2 (MMPOWER-1) [9] and Phase 2 (MMPOWER-2) [10] clinical trials, in which treatment with elamipretide was analyzed in patients with PMM. Genetic variants within the MMPOWER-3 trial population (i.e., both mtDNA and nDNA) have previously been published, along with the finding that subjects with nDNA pathogenic variants who received elamipretide performed significantly better on the 6MWT in the trial compared with placebo [6]. Although MMPOWER-3 trial did not meet its primary endpoints, post hoc analysis of results by genetic subtype have emphasized the importance of considering specific disease genotypes and phenotypical presentation in the design of interventional clinical trials. As previously published, the *post-hoc* genetic subgroup analysis on the co-primary endpoint in MMPOWER3, Total Fatigue Score on the Primary Mitochondrial Myopathy Symptom Assessment (PMMSA TFS), did not demonstrate a differential effect when the nDNA and mtDNA cohorts were compared [6]. The reason a significant differential effect with daily elamipretide was seen between the nDNA and mtDNA cohorts in 6MWT and not with the PMMSA TFS outcome measure is not known. Fatigue is known to be a significant burden for many patients with PMM; however, the different types or components of fatigue contributing to overall fatigue in patients is not well understood and was not differentiated in the trial.

This manuscript presents new analyses and highlights novel findings of interest to the field. First, there was significant improvement and a differential response in 6MWT in subjects with mtDNA replisome pathogenic variants, an exciting finding that may help enrich future interventional studies in PMM. Second, the significant placebo effect in individuals with *MT-TL1* pathogenic variants profoundly influenced the overall results of the MMPOWER-3 trial given the relatively high proportion of subjects with this mtDNA genotype in the trial. Although the factors that led to this placebo effect are not fully understood, variability among this mtDNA cohort appears to have contributed. A number of individuals with low heteroplasmy in *MT-TL1* and randomized to placebo walked farther at this timepoint, which greatly contributed to the observed placebo effect. Third, an exposure–response relationship in the nDNA cohort suggested a weak (albeit significant) positive correlation between plasma elamipretide levels and pharmacodynamic response in the 6MWT. These data were used as a partial justification for increasing to a 60 mg dose in NuPOWER. Finally, based on these data, a follow-up trial has been designed and initiated with a more specific trial population, an enrichment strategy that may increase the likelihood for success in treating PMM [11].

The mtDNA replisome pathogenic variant subgroup contained genes responsible for mtDNA replication and maintaining the mitochondrial nucleotide pool. Our analyses revealed no placebo effect in this cohort, which was reassuring and consistent with placebo arms from earlier trials using elamipretide [9, 10].

The majority of subjects in the mtDNA replisome cohort had pathogenic variants in *POLG*, the most commonly affected nuclear gene in the North American Mitochondrial Disease Consortium Registry [12]. Although still rare, *POLG* is a nuclear gene that encodes the sole mitochondrial DNA polymerase enzyme. *POLG* pathogenic variants are among the more common causes of inherited mitochondrial diseases [13]. The *POLG* enzyme contains proof-reading, polymerase, and linker domains, making this enzyme important for both replication and fidelity of mtDNA copies [14]. Our analyses revealed that individuals with *POLG* pathogenic variants responded similarly to the mtDNA replisome cohort as a whole, and elamipretide did not appear to discriminate between the locus of *POLG* pathogenic variants and the improvement in 6MWT in the trial (data not shown). *POLG* pathogenic variants were seen across the endonuclease, linker, and polymerase regions of the enzyme, and represented similarly between the elamipretide and placebo-treated groups.

The prevalence of *POLG* pathogenic variants in the overall Phase 3 MMPOWER-3 trial was roughly 13% of the population (majority being monoallelic, causing dominant disease), within the previously-reported range of 4% to 26% across various studies [13, 15, 16]. *POLG* pathogenic variants lead to a continuum broad spectrum of clinical features that can present at any age; however, age at disease onset can provide information regarding diagnosis and outcome. For example, the onset of CPEO dominates the *POLG* clinical spectrum in older patients (> 40 years); occipital epilepsy tends to occur in younger patients (< 12 years); and peripheral neuropathy and ataxia most often occurs between 12 and 40 years of age [17]. Notably, our results suggest that CPEO involvement was associated with greater clinical benefit of elamipretide, suggesting certain nDNA phenotypes (i.e., adult-onset myopathies in patients > 40 years of age) may be more likely to respond to treatment with elamipretide. Similar improvements were observed in individuals with *TWNK* pathogenic variants, all of whom had CPEO.

Interestingly, the clinical trial results may also advance our mechanistic insight of targeting cardiolipin with elamipretide in PMM. mtDNA replication is essential for maintaining energy homeostasis, and there is a direct correlation between mtDNA copy number and the biosynthesis of the mitochondrial respiratory chain enzyme complexes [18]. As previously described, all of the enzymes responsible for mtDNA maintenance encoded by nDNA are synthesized in the cytoplasm [6], and therefore must be transported across the inner mitochondrial membrane, which is enriched with cardiolipin [6, 19–21]. Metabolite and nucleotide transporters depend on cardiolipin, the signature phospholipid of the mitochondrial inner membrane, for their assembly and activity [6, 22]. Cardiolipin is also known to stabilize mtDNA packaging into nucleoids, providing maintenance of mtDNA integrity and respiratory function [23]. Elamipretide is hypothesized to affect the mtDNA replisome, at least partly, via a reduction in the leak of reactive oxygen species (ROS) by helping to colocalize electron transport complexes. Since mtDNA replisome components are packaged into mitochondrial nucleoids that are in close proximity to the electron transport chain [24], the mtDNA replisome is likely susceptible to ROS produced in close proximity to the electron transport chain [25]. In addition, since elamipretide stabilizes cardiolipin [26], elamipretide may enhance cardiolipin-dependent functions including inner mitochondrial membrane protein import/assembly, metabolite/nucleotide transport, and mtDNA stability. These presumptions are supported by preclinical work in which elamipretide improved various aspects of mitochondrial function and morphology [23, 27–30].

Pharmacokinetic analyses in the nDNA cohort also showed a trend among subjects with higher elamipretide exposure (measured in plasma) and improved 6MWT. These data are encouraging and implicate a possible pharmacokinetic-pharmacodynamic relationship in this cohort.

Taken together, these data have provided the foundation for a subsequent Phase 3 clinical trial enriched with this population and using a 60 mg dose of elamipretide (depicted in supplemental Fig. 6), which has been initiated and fully enrolled at this time (NuPOWER Clinical Trial, SPIMD-301, NCT05162768) [11]. NuPOWER was designed to evaluate the efficacy and tolerability of elamipretide in nPMD subjects, with the primary efficacy endpoint being distance walked (meters) on the 6MWT [11]. Elamipretide was also studied in subjects with Barth Syndrome (TAZPOWER, SPIBA-201, NCT03098797), which is an X-linked mitochondrial disease caused by defects in *TAZ*, a gene responsible for cardiolipin remodeling [31]. After approximately 36-weeks in the 168-week open-label phase, elamipretide was associated with significant and consistent improvements in 6MWT (n = 8, 95.9 m, *p* = 0.02) and BTHS–SA TFS [31]. There were also significant improvements in secondary endpoints including knee extensor strength (skeletal muscle), patient global impression of symptoms, and some cardiac parameters (specifically stroke volume and cardiac output) [31].

**Fig. 6:**
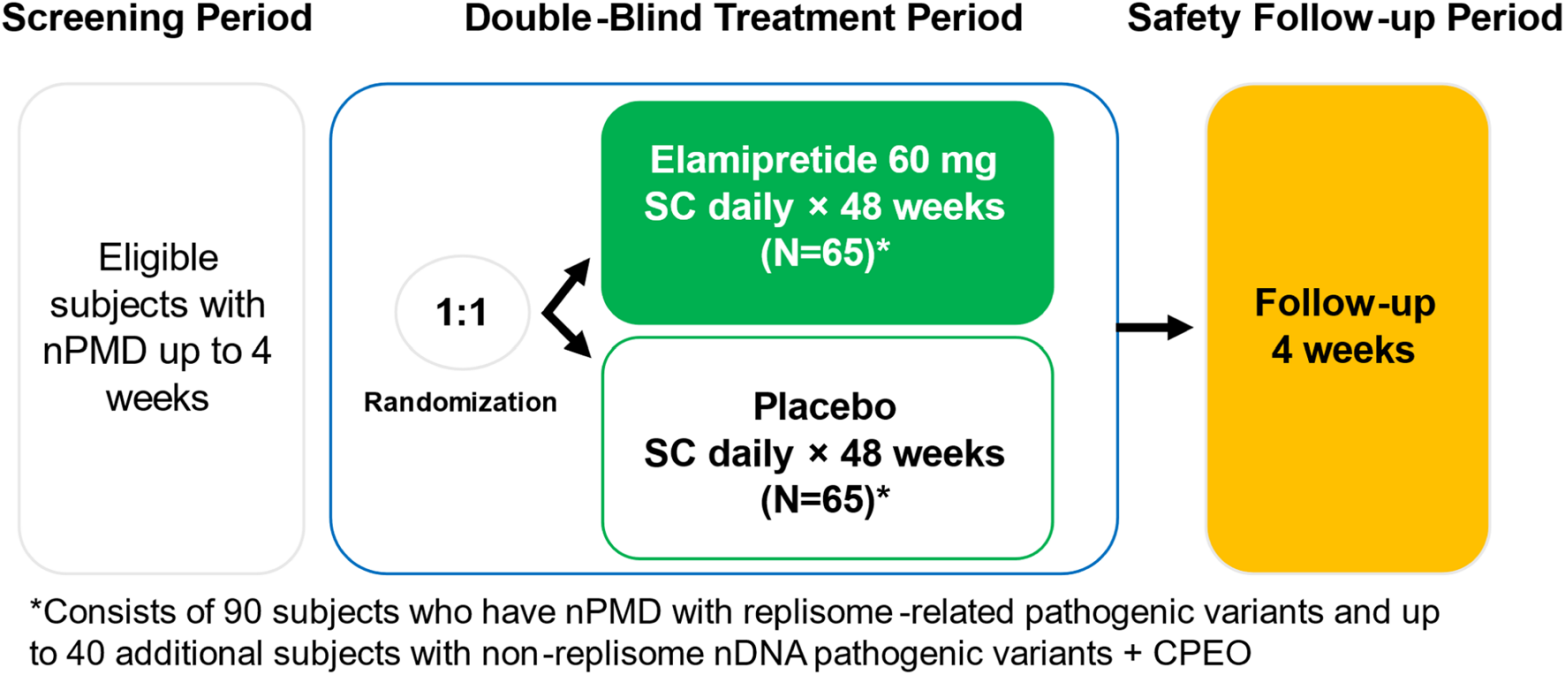
Phase 3 trial design of NuPOWER enrolling subjects with replisome-related nDNA pathogenic variants and CPEO^10^

Another consequence of the analyses presented here is a better understanding of the genotype-specific responses in the mtDNA alteration cohort. The prominent placebo effect in the MMPOWER-3 trial [6] was unexpected and not predicted by the Phase 2 trial (MMPOWER-2) [9]. The mtDNA cohort accounted for about three-quarters of the subjects within the overall Phase 3 trial [6]. The majority of these subjects (approximately 70%) had either single large-scale mtDNA deletions or pathogenic variants in *MT-TL1.*

There are several limitations that must be acknowledged. Primary mitochondrial disease is both genetically and phenotypically heterogenous. We have previously acknowledged that “basket” trial designs may induce insurmountable heterogeneity in rare disease clinical trials [6], leading to cautious optimism from our post hoc genotype analysis in this small cohort of individuals. Furthermore, the 6MWT was the primary endpoint examined in the subgroup analysis and the only measure to demonstrate a strong differential effect relative to the nDNA and mtDNA cohorts. The lack of differences in other endpoints and the existence of helpful (but not definitive) and universally accepted biomarkers in adults with PMM also leave room for caution. The ongoing work to further understand the genotype/phenotype relationship within the heterogeneous family of mitochondrial disease, the emergence of additional objective endpoints (eg, Mitochondrial Myopathy-Composite Assessment Tool [32]), reliable biomarkers, and predictive pre-clinical models will all strengthen the design of interventional clinical trials and bolster PMM treatments in the years ahead.

## Conclusions

This analysis suggests that elamipretide has a beneficial effect on ambulatory exercise capacity in patients with PMM with nuclear gene-encoded mtDNA replisome disorders. The data highlight the importance of considering genetic subtypes in PMM. The benefit was particularly relevant in those with replisome pathogenic variants and CPEO. These findings emphasize the challenge of developing therapies for the broadly heterogeneous class of mitochondrial diseases and reinforce the importance of focusing on genetic subgroups when developing treatments for individuals with PMM, as well as providing insights into various genetic abnormalities and the likelihood of responding to elamipretide for patients with PMM. Based on the observations from this post hoc analysis, a trial to evaluate the efficacy and safety of elamipretide in subjects with primary mitochondrial disease resulting from nDNA mutations (NuPOWER) was designed and is now fully enrolled [11].

## Data Availability

The datasets supporting the conclusions of the *post-hoc* analysis described in this article are included within the article. Data from the MMPOWER-3 study and the *post-hoc* analysis not published within this article will be made available by request from the corresponding author. Full datasets from the MMPOWER-3 clinical trial are available at: https://clinicaltrials.gov/study/NCT03323749?tab=results. Anonymized data not published within this article will be made available by request from any qualified investigator. Additional data may also be found at: Study Record | Beta ClinicalTrials.gov (NCT NCT02976038).

## Appendix 1

See Table

**Table 1:** MMPOWER-3 Trial Co-investigator Members

Name	Affiliation
Valentino M.L	IRCCS Istituto delle Scienze Neurologiche di Bologna, Programma di Neurogenetica, Bologna, Italy, and Department of Biomedical and Neuromotor Sciences, University of Bologna, Bologna, Italy
Primiano G	Fondazione Policlinico Universitario A. Gemelli and Istituto di Neurologia, Università Cattolica del Sacro Cuore, Rome, Italy
Sancricca C	
Grosz Z	Institute of Genomic Medicine and Rare Disorders, Semmelweis University, Budapest, Hungary
Diodato D	Bambino Gesù Ospedale Pediatrico, IRCCS, Rome, Italy
Vasco G	
Soler-Alfonso C	Baylor College of Medicine and Texas Children’s Hospital
Ali M	
Hanna M.G	Department of Neuromuscular Diseases, UCL Queen Square Institute of Neurology and The National Hospital for Neurology and Neurosurgery, London, UK
Bugiardini E	
Poole O.V	
Kendall F	Rare Disease Research, Atlanta, GA, USA
Larson A	University of Colorado and Children’s Hospital Colorado, Aurora, CO, USA
Nithi A	Columbia University Irving Medical Center, New York, NY, USA
Engelstad K	
Mattman A	Vancouver General Hospital, Vancouver, BC, Canada
Mezei M	
Bischoff A.T	Friedrich-Baur-Institute, Department of Neurology, LMU Hospital, Ludwig Maximilian University of Munich, Ziemssenstraße 1a, 80336, Munich, Germany
Büchner B	
Radelfahr F	
Stendel C	
Catarino C.B	
Steiner S	Rebecca D. Considine Research Institute, Akron Children’s Hospital, Akron, OH, USA
Rossman I	
Cole K	
Victorio M.C	
Montano V	Department of Clinical and Experimental Medicine, Neurological Institute, University of Pisa, Italy
Lopriore P	
Siciliano G	
Musumeci O	Neurology and Neuromuscular Unit, Department of Clinical and Experimental Medicine, University of Messina, Messina, Italy
Catania A	Fondazione IRCCS istituto Neurlogico C. Besta Milano

1.

## Abbreviations

6MWT: Six-minute walk test
ATP: Adenosine triphosphate
AUC: Area under the plasma concentration-time curve
CPEO: Chronic progressive external ophthalmoplegia
LS: Least Squares
MDDS: MtDNA depletion and deletions syndrome
MMRM: Mixed model repeated measures
mtDNA: Mitochondrial DNA
nDNA: Nuclear DNA
nPMD: Nuclear primary mitochondrial disease
PMM: Primary mitochondrial myopathy
PMMSA TFS: Total fatigue score on the primary mitochondrial myopathy symptom assessment
POLG: Polymerase gamma
r: Correlation coefficient
ROS: Reactive oxygen species
TWNK: Twinkle
